# Microstructural Evolution and Strength Development of High-Water-Content Soft Soils Stabilized with Cementitious–Expansive Binders

**DOI:** 10.3390/ma19132828

**Published:** 2026-07-02

**Authors:** Youmin Han, Yunlong Zhao, Beiping Han, Li Jiang, Hongfei Chang, Junwu Xia

**Affiliations:** 1 School of Architecture and Civil Engineering, Anhui Polytechnic University, Wuhu 241000, China; hanyoumin@ahpu.edu.cn (Y.H.); 2231422102@stu.ahpu.edu.cn (Y.Z.); 2241422107@stu.ahpu.edu.cn (B.H.); jl@ahpu.edu.cn (L.J.); 2 Anhui Engineering Research Center of Green Building and Digital Construction, Anhui Polytechnic University, Wuhu 241000, China; 3 State Key Laboratory of Intelligent Construction and Healthy Operation and Maintenance of Deep Underground Engineering, China University of Mining and Technology, Xuzhou 221116, China; honfee@126.com; 4 Jiangsu Key Laboratory of Disaster Prevention and Intelligent Control in Civil Engineering, School of Mechanics and Civil Engineering, China University of Mining and Technology, Xuzhou 221116, China

**Keywords:** high-water-content soft soil, soil stabilization, composite stabilizer, ettringite (AFt), microstructural mechanism

## Abstract

This study experimentally investigated the stabilization mechanisms and structure formation models of high-water-content soft soils (>70%) treated with ordinary Portland cement, sulfur aluminate cement, gypsum, and lime. Fifteen single- and composite-stabilizer systems were evaluated using unconfined compressive strength (UCS) tests and microstructural analyses, including SEM, XRD, TG–DTG, and FTIR analyses. The results show that stabilized soils containing cementitious components exhibit significantly higher strength due to the formation of calcium silicate hydrate (C–S–H) gel, which effectively binds soil particles. The addition of sulfur aluminate cement, gypsum, and lime promotes rapid hydration and generates abundant ettringite (AFt) crystals with strong water absorption capacity, contributing to early strength development. Based on these findings, a composite stabilizer (ECS) combining cement with appropriate proportions of sulfur aluminate cement, gypsum, and lime is proposed, achieving substantial improvements in both early and long-term strength. The stabilization process proceeds in two stages: rapid AFt formation absorbs free water and fills large pores to form a three-dimensional network, and then C–S–H gel cementation integrates the soil–AFt framework into a dense and coherent structure. The study provides mechanistic insight and a theoretical basis for stabilizing high-water-content soft soils in coastal and riparian engineering applications.

## 1. Introduction

Soft soils, commonly defined as soils with a natural water content exceeding 30% or higher than their liquid limit, are widely distributed in coastal and riparian regions and are characterized by low shear strength, high compressibility, and poor bearing capacity [1]. In their natural state, such soils generally fail to satisfy engineering performance requirements and therefore require stabilization before being used as foundation or fill materials [2]. While existing studies and engineering practice indicate that soft soils with water contents of approximately 30–70% can achieve acceptable mechanical performance after appropriate stabilization, rapid urbanization and infrastructure development in coastal and riparian zones have increasingly exposed the limitations of current approaches when applied to soft soils with extremely high water contents reaching up to 100%. These soils are particularly prevalent in coastal tidal flat areas, making the stabilization and reinforcement of high-water-content soft soil foundations a critical scientific and engineering challenge for the sustainable development of coastal infrastructure.

Compared with conventional soft soils, high-water-content soft soils exhibit fundamentally different mechanical behavior and stabilization responses due to their excessive pore water, weak soil skeleton, and limited particle interlocking. Understanding the hardening and strength development mechanisms of these soils is therefore essential for developing effective stabilization strategies.

Up to now, studies have tried to improve the mechanical properties of soft soils with high water content by using curing agents. Usually, cement and lime are used as the main cementing materials, in addition to some industrial waste such as fly ash, blast furnace slag, recycled glass, etc., and sometimes bentonite, silica, epoxy resin, and redispersible polymers, which are used to improve the curing effect [3]. Ground granulated blast furnace slag (GGBS), a by-product from the iron industry, has attracted enormous attention in treating high-water-content soils due to its sustainability advantages and good performance in reducing swelling [4], but it has to be chemically activated to form cementitious products, e.g., calcium silicate hydrate (C–S–H) [5,6]. Lime (i.e., quicklime and hydrated lime) is a frequently used GGBS activator in soil stabilization due to its low cost and good availability [7,8]. As an additive, GGBS has a significant effect on curing various soils such as soft soils with high water content and highly plastic clays [4,9,10,11,12], but it still needs to be used with some basic curing agents such as cement and lime. Fly ash, glass fiber, construction waste residues, and other curing agents [13,14,15] cannot completely replace these foundational curing materials when curing soft soils with high water content.

There has been a lot of research on the curing of high-water-content soils with basic materials such as cement, gypsum, and lime. Cement and gypsum-reinforced soft clay generally has higher strength than cement alone, and saves about 25% of the cement, but the curing effect is not ideal for soft soils with high water content [16]. Han found that higher compressive strength could be observed in soil stabilized by the combination of cement, gypsum, and lime, in contrast to that with single- or dual-stabilizer mixtures. Sufficient C–S–H gel-cemented soil particles can be formed after the complete hydration reaction of cement, and the addition of the lime and gypsum hydration reaction produces calcite crystals to fill the pores between the soil particles [17]. Relevant studies have also shown that sulfur aluminate cement, gypsum, and lime can form high-water fast-setting materials, which have the characteristics of “high water” and “rapid setting”, and, when they are configured according to a 5:4:1 ratio, the hydration reaction can generate sufficient calcium crystals to fill the pores between soil particles [18,19].

Although previous studies have shown that stabilizers can enhance the strength of high-water-content soft soils, a clear mechanistic understanding remains lacking. Existing research largely focused on macroscopic strength improvement, with limited insight into the coupled chemical–microstructural processes governing hardening under extremely high water contents. Moreover, systematic comparative studies using fundamental and widely available cementitious materials are scarce, and the relationship between hydration product evolution (e.g., C–S–H gels and ettringite) and strength development has not been fully clarified. Structural formation models describing stabilized high-water-content soft soils are also largely absent.

To address these gaps, this study experimentally explores the stabilization performance of high-water-content soft soils with Portland cement, sulfur aluminate cement, gypsum, and lime. Fifteen representative mix proportions are designed to reveal the evolution characteristics of hydration products including C–S–H gel and ettringite. Strength tests combined with microstructural analysis are conducted to elucidate hardening mechanisms and propose structure formation models, which provide a theoretical basis for ground improvement in coastal and riverside areas.

## 2. Materials and Methods

### 2.1. Materials

The soil used in the experiments was collected from the third clay layer of the foundation pit at the Undergraduate Innovation and Training Center construction site on the Nanhu Campus of the China University of Mining and Technology. The original clay was air-dried, crushed, sieved through a 2 mm sieve, and finally sealed in bags for use as the test soil. The main mineral components of the test soil included quartz, kaolinite, illite, and montmorillonite. The characteristic indices of the test soil were as follows: pH value: 7.92, specific gravity: 2.75, plastic limit: 24.1%, and liquid limit: 44.2%. In terms of particle size distribution, clay (<0.002 mm) accounted for 9.15%, silt (0.002–0.075 mm) accounted for 80.28%, and sand (0.075–2 mm) accounted for 10.57%.

The raw materials used as curing agents in the experiments included P.O 42.5 cement (C), lime (L), gypsum (G), and sulfur aluminate cement (SA). The main components of ordinary Portland cement are 3CaO·SiO_2_, 2CaO·SiO_2_, 3CaO·Al_2_O_3_, 4CaO·Al_2_O_3_·Fe_2_O_3_, and 3CaSO_4_. The main components of sulfur aluminate cement are 3CaO·3Al_2_O_3_·CaSO_4_, 2CaO·SiO_2_, and 4CaO·Al_2_O_3_·Fe_2_O_3_. Table 1 presents the mass percentages of various oxide constituents of the cement, lime, gypsum, and sulphoaluminate cement obtained via XRF testing.

### 2.2. Sample Preparation

According to the Standard for Geotechnical Test Methods (GB/T 50123) [20], the masses of the test soil, each component of the curing agent, and the water were weighed according to the designed proportions. First, the weighed test soil and each component of the curing agent were poured into a mixing pot and mixed evenly by hand. Then, the weighed water was added, and the mixture was stirred uniformly using a mixer. When the stabilized soil slurry reached a certain viscosity, it was poured into 50 × 50 × 50 mm^3^ cubic molds that had been coated with petroleum jelly as a release agent. The slurry was compacted and formed on a small vibration table for three minutes, with 3 specimens prepared per group. Excess stabilized soil slurry on the top surface of the molds was scraped off and leveled, and a label with the corresponding number was attached to the surface of each specimen before they were covered with plastic film. During the specimen casting process, the indoor environment temperature and water temperature were approximately 23 °C and the relative humidity was about 72%. After 24 h, all test pieces were demolded and wrapped by plastic film. Curing was carried out in a standard room with a temperature of 20 ± 2 °C and a relative humidity of 95 ± 5% up to the target curing age.

### 2.3. Methods

#### 2.3.1. Unconfined Compressive Strength (UCS) Tests

In this study, the unconfined compressive strength (UCS) tests were conducted using a 50 kN microcomputer-controlled electronic universal testing machine (model: WDW-50) which is manufactured by Jinan Dongce Testing Machine Technology Co., Ltd. (Jinan, China). The loading rate was set to 1 mm/min, and the average UCS value of three specimens was taken as the compressive strength for each group.

From each group of crushed specimens, one specimen was selected and immersed in absolute ethanol for 7 days to terminate the hydration reaction, which was subsequently used for scanning electron microscope (SEM), X-ray diffraction (XRD), thermogravimetric and derivative thermogravimetric (TG-DTG), and Fourier transform infrared spectrometer (FTIR) tests.

#### 2.3.2. Microscopic Study

To investigate the hydration and hardening reaction mechanisms of each calcium-based stabilized soil, microscopic testing methods were used to characterize the hydration products and microstructural features of the calcium-based stabilized soils. Microscopic testing methods such as SEM, XRD, TG-DTG, and FTIR exhibit varying sensitivities in the characterization of different phases. A single testing method cannot yield valid conclusions. However, the test results from different methods can be mutually supplemented and cross-validated to determine the hydration products and microstructural features of each type of stabilized soil. By comparing and analyzing the strength, hydration products, and microstructural features of each calcium-based stabilized soil, the mechanisms by which hydration products and microstructural features influence the strength of calcium-based stabilized soil can be revealed.

In this paper, a Quanta^TM^ 250 scanning electron microscope (SEM) manufactured by the FEI Company (Hillsboro, OR, USA) was used for analysis. The samples were first dried in an oven at 60 °C and then cut into small pieces of 7 × 7 × 7 mm^3^. After exposure of the fresh surfaces, they were fixed onto the sample holder with double-sided tape and subjected to gold sputtering treatment to improve conductivity. Finally, images were collected under high-vacuum mode at a magnification of 8000 times.

XRD analysis was performed using a D8 ADVANCE X-ray diffractometer produced by the Bruker Company of Ettlingen, Germany. After being dried at 60 °C, the samples were crushed and ground into powder and sieved through a 200-mesh sieve, and at least 1 g of powder was collected for XRD analysis.

TG analysis was carried out using a TGA/DSC1/1100LF synchronous thermal analyzer from the Mettler Toledo Company of Greifensee, Switzerland. The DTG curve reflects the relationship between the rate of weight change and temperature, and it is the first derivative of the TG curve. The sample preparation method was the same as before, and no less than 0.5 g of powder was taken from each group for testing.

A VERTEX 80v Fourier transform infrared spectrometer (FTIR) produced by Bruker (Ettlingen, Germany) was used for analysis. The FTIR forms a characteristic absorption spectrum through the resonance between molecular groups and infrared light, which is used to infer the molecular structure and phase content. The sample preparation method was the same as before, and no less than 1 g of powder was taken from each group for testing.

#### 2.3.3. Study of Cementitious–Expansive Binders for Soft Soil Stabilizers

The representative tests in this study included 15 groups of stabilized soil specimens with cement (C), lime (L), gypsum (G), and sulfur aluminate cement (SA) as the raw materials for the stabilizers. Among these groups, there was 4 groups with a single stabilizer, 6 groups with two mixed stabilizers, 4 groups with ternary mixed stabilizers, and 1 group with quaternary mixed stabilizers. Naming rule example: C70G30S represents the solidified soil prepared with a mix proportion of 70% C and 30% G.

Details regarding the grouping scheme of the stabilizer representative tests, the composition of each stabilizer raw material, and their mass percentages are provided in Table 2. The stabilizer dosage for all stabilized soil specimens was 16%, the designed moisture content of the soft soil was 80%, and the curing age was 28 days.

The water content of soft soil mentioned in this paper refers to the total water content, namely, the ratio of the mass of all water (including water in the original soil and curing agent) to the mass of oven-dried soil.

Specimens of each group were prepared and cured to the curing age in accordance with the test grouping scheme described in Table 2 and Section 2.2. After the stabilized soil specimens were cured to the curing age, UCS and microscopic tests were conducted sequentially in accordance with the test and testing methods specified in Table 3.

## 3. Results

### 3.1. Unconfined Compressive Strengths of Stabilized Soils with Different Binder Compositions

**Figure 1:**
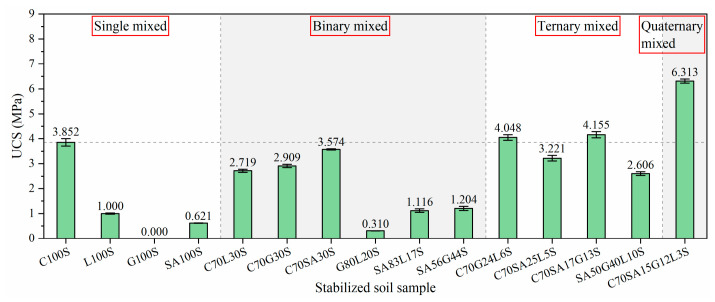
Unconfined compressive strengths of stabilized soils with different binder compositions.

The ultimate strength from the stress–strain curve of each stabilized soil is taken as the unconfined compressive strength of the corresponding stabilized soil, and the unconfined compressive strength values of all stabilized soils obtained are shown in Figure 1.

In accordance with relevant findings from the existing literature [17], in single-component stabilized soils, G100S fails to form a shape even after a 28-day curing period, and thus has no measurable compressive strength. In contrast with C100S, the compressive strength of L100S is just 25.96%. Among the specimens stabilized by a single type of additive, C100S possesses superior strength compared to L100S, while G100S cannot form a stable shape. For binary mixed stabilized soils, the compressive strengths of the C70L30S and C70G30S are 70.59% and 75.52% of that of the C100S, respectively. This shows that the composite curing agents formed by adding lime or gypsum individually to cement do not enhance the compressive strength of the stabilized soil. In addition, the compressive strength of G80L20S is even lower, accounting for 8.05% of that of C100S.

In this study, focusing on ternary stabilized soils, the compressive strengths of C70G24L6S, C70SA25L5S, C70SA17G13S, and SA50G40L10S are 105.09%, 83.62%, 107.87%, and 72.59% of the compressive strength of C100S, respectively. Among these mixtures, C70G24L6S and C70SA17G13S exhibit compressive strengths slightly higher than that of C100S, C70SA25L5S shows a slightly lower compressive strength, and SA50G40L10S exhibits a substantially lower strength.

In the quaternary stabilized soil system, the compressive strength of C70SA15G12L3S (ECSS) is 162.18% of that of the C100S, which is significantly higher than the compressive strength of the C100S. This indicates that the composite curing agent formed by simultaneously adding sulfur aluminate cement, gypsum, and lime to cement at an appropriate ratio can substantially improve the compressive strength of the stabilized soil.

From the comparative analysis of the 15 types of stabilized soils (including single, binary, ternary, and quaternary mixed systems), it can be concluded that all stabilized soils containing cementitious components have relatively high compressive strength, while those without cementitious component have relatively low compressive strength. This indicates that the cementation effect of C-S-H gel plays a crucial role in improving the compressive strength of stabilized soils, and the cementitious component is indispensable in the composition of curing agents.

### 3.2. Microscopic Analysis Results

#### 3.2.1. SEM Results

**Figure 2:**
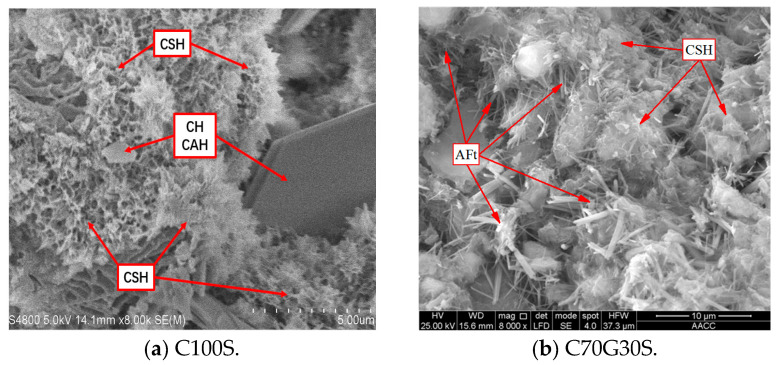

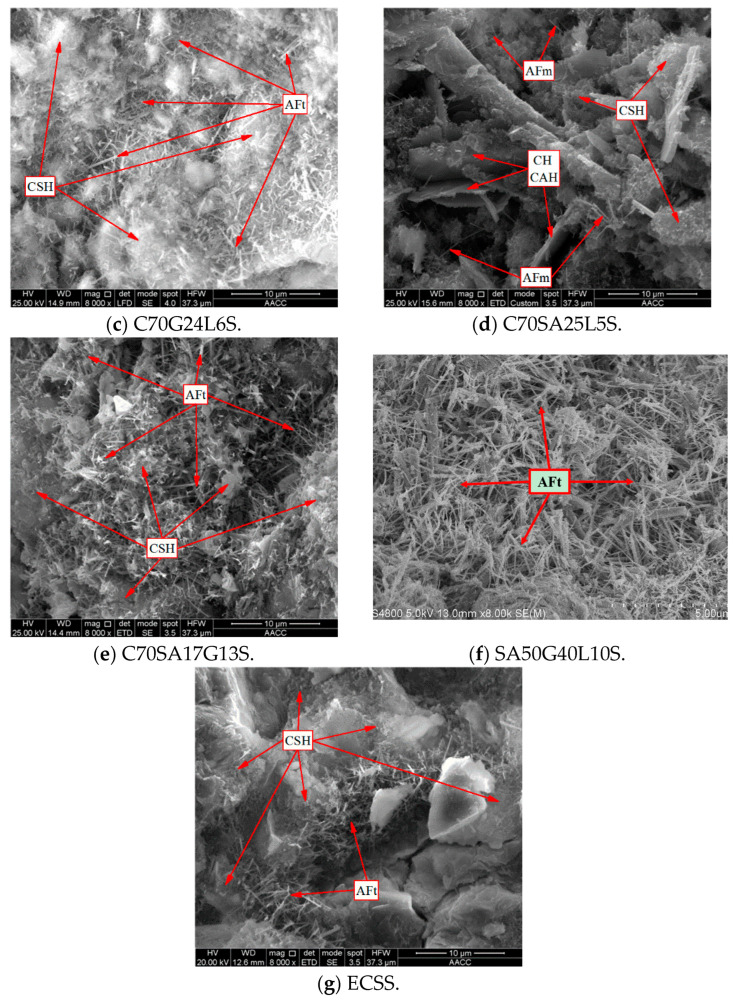
The microscopic test results of SEM.

As can be seen from Figure 2a, a large number of flocculent C–S–H gels are formed in the C100S [21,22,23,24] which wrap around the outer surface of soil grains and cement the soil grains together to form an integrated mass, although some relatively large pores are still visible. Trace amounts of acicular AFt crystals and AFm crystals can be observed in the pores [21,22,24,25], interspersed with a few hexagonal platy CH crystals [26,27,28,29] and cubic CAH crystals. Among these, the AFt crystals have a length of approximately 1–2 μm and a diameter of about 0.1 μm, and their quantity is insufficient to fully fill the larger pores.

Figure 2b shows that a large number of flocculent C–S–H gels and a substantial quantity of acicular–columnar AFt crystals [25] are formed in the C70G30S, while no obvious hexagonal platy CH crystals or cubic CAH crystals are observed. The AFt crystals have a length of approximately 2–5 μm and a diameter of about 0.1–0.5 μm, and their size is significantly larger than that of the AFt crystals in the C100S. Due to the significant difference in scale between the C–S–H gels and the AFt crystals, the two are largely independent of each other. An excessive number of thick and long AFt crystals expand the pores between soil grains even further, while the C–S–H gels are unable to cement the soil grains and AFt crystals together to form an integrated mass, resulting in a relatively loose overall structure.

As depicted in Figure 2c, a large number of flocculent C–S–H gels and a relatively high content of acicular AFt crystals are formed in the C70G24L6S. The observed AFt crystals present a length of 1–3 μm and a diameter of 0.1–0.2 μm. In terms of particle size, they exceed the AFt crystals in C100S slightly while being considerably smaller than those in C70G30S. Microscopic morphology observations indicate that, by filling large gaps between soil particles and cross-linking with one another, these crystals construct a spatial network and reduce pore dimensions. Furthermore, C–S–H gels cover soil grains and the AFt network framework. Under the cementation of C–S–H gels, soil particles and AFt networks are integrated into a relatively dense solid body.

As can be seen from Figure 2d, a large number of flocculent C–S–H gels, CH crystals, and CAH crystals and a small number of acicular–columnar AFm crystals are formed in the C70SA25L5S. Microscopic morphology observations indicate that the small number of AFm crystals fill the large pores between soil grains, while the CH crystals and CAH crystals further enlarge the pore space. The C–S–H gels wrap around the outer surfaces of the AFm crystals, CH crystals, and CAH crystals.

Figure 2e shows that a relatively large number of flocculent C–S–H gels and an extremely high quantity of acicular–columnar AFt crystals are formed in the C70SA17G13. The AFt crystals have a length of approximately 2–5 μm and a diameter of about 0.1–0.5 μm, a size which is comparable to that of the AFt crystals in the C70G24L6S. The large gaps between soil particles are filled with AFt crystals that interlink into a network. C–S–H gels adhere to the surface of soil grains and this AFt network, thus consolidating the system into an intact entity. As depicted in Figure 2f, an extremely large number of acicular AFt crystals are formed in the SA50G40L10S. The AFt crystals have a length of approximately 1–3 μm and a diameter of about 0.1–0.2 μm. Microscopic morphology observations indicate that the large gaps between soil particles are filled by AFt crystals, which interweave to establish a complete spatial network. However, since no C–S–H gel is observed to cement the soil grains and the AFt network, the overall structure remains relatively loose and porous.

As can be seen from Figure 2g, a large number of flocculent C–S–H gels and a relatively high content of acicular AFt crystals are formed in the ECSS. The AFt crystals have a length of approximately 1–3 μm and a diameter of about 0.1–0.2 μm, and their size is similar to that of the AFt crystals in the high-water-content material-stabilized soil. The large pores between soil particles are filled with AFt crystals. Their mutual interweaving constructs a spatial network and makes the pores relatively narrow. In addition, C–S–H gels attach to the surface of soil grains as well as the three-dimensional network formed by AFt crystals. The cementing reaction induced by C–S–H gels effectively binds soil particles and AFt network frameworks into a dense and integrated solid structure. In many microscopic regions, C–S–H coating fills the gaps between AFt crystals, rendering pores invisible.

#### 3.2.2. XRD Results

XRD is a common microscopic characterization technique in civil and material engineering. After baseline correction of raw XRD data, mineral phases and hydration products of specimens are identified by matching diffraction peaks with standard PDF cards, and semi-quantitative analysis of relative mineral content is performed using diffraction peak parameters [30].

**Figure 3:**
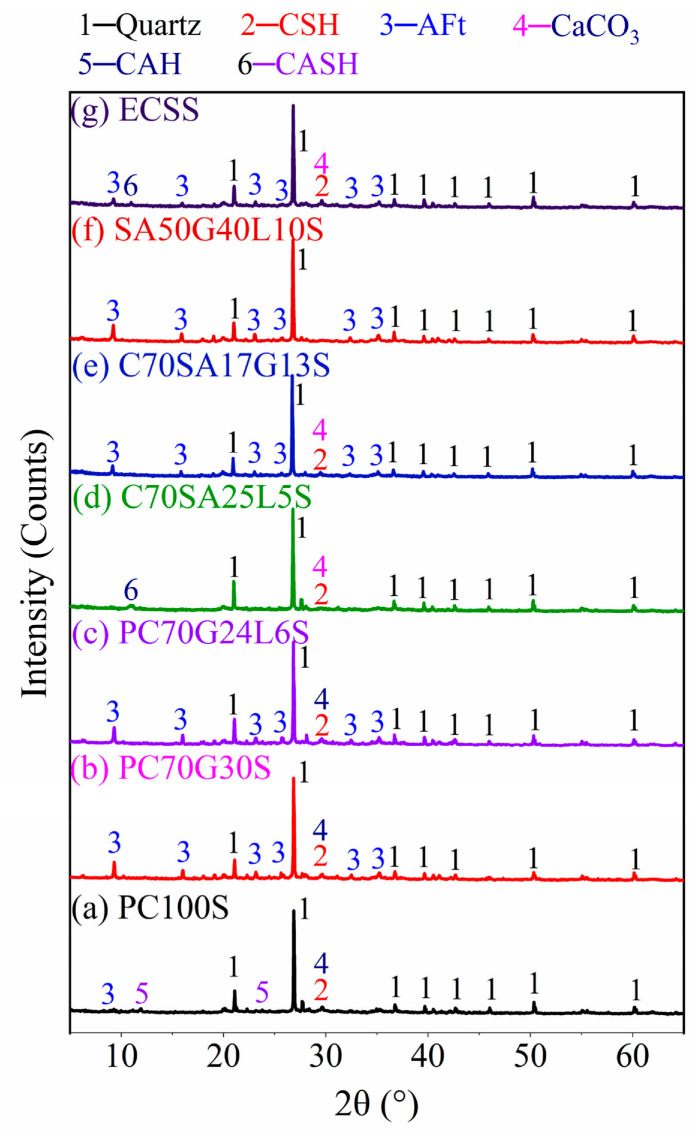
The microscopic test results of XRD.

In Figure 3, C100S exhibits weak C–S–H gel peaks and CaCO_3_ peaks, as well as faint AFt crystal peaks and CAH crystal peaks [22,31]. Due to the poor crystallinity of C–S–H gel, its low XRD peak intensity does not necessarily indicate a low production quantity. To determine the production amount of C–S–H gel in the sample, a comprehensive analysis using other testing methods such as SEM and TG-DTG is also required. The sample C70G30S displays highly intense peaks for AFt. By contrast, the signals from C–S–H gels and CaCO_3_ are weak [25]. The C70G24L6S presents extremely strong AFt crystal peaks, as well as weak C–S–H gel peaks and CaCO_3_ peaks. According to the XRD results alone, there is little difference between C70G24L6S and C70G30S, and a comprehensive analysis combined with SEM observations is still needed. The C70SA25L5S has faint CASH (zeolite-like phase) gel peaks [32], C–S–H gel peaks, and CaCO_3_ peaks. The C70SA17G13S exhibits strong AFt crystal peaks, along with weak C–S–H gel peaks and CaCO_3_ peaks. The SA50G40L10S shows extremely strong AFt crystal peaks, while no C–S–H gel peaks are observed. The ECSS presents relatively strong AFt crystal peaks and faint CASH (zeolite-like phase) gel peaks [32], as well as weak C–S–H gel peaks and CaCO_3_ peaks.

#### 3.2.3. TG-DTG Results

TG-DTG analysis is an effective thermal analysis technique widely adopted to evaluate the thermal stability, component composition, and thermal decomposition behavior of engineering materials. The TG curve reflects the continuous mass loss of specimens during programmed temperature elevation, which can be utilized to quantify the mass loss ratio and residual mass corresponding to different temperature intervals. As the differential form of the TG curve, the DTG curve precisely characterizes the thermal decomposition rate and determines the peak temperature and reaction intensity of each weight-loss stage. Combined analysis of TG and DTG curves enables the identification of various thermal response stages, including free water evaporation, dehydration and decomposition of hydration products, and thermal degradation of matrix phases [33].

**Figure 4:**
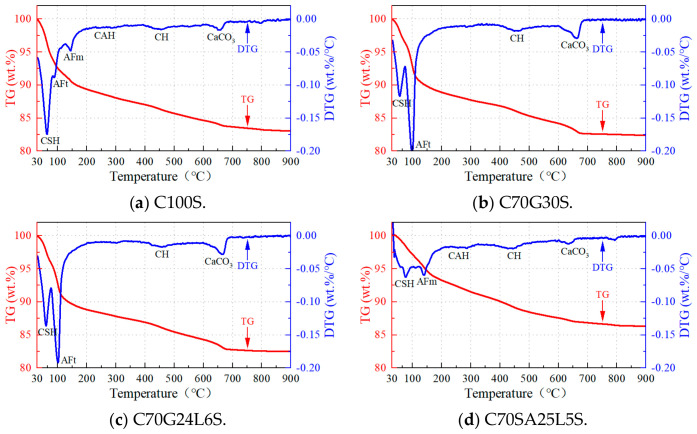

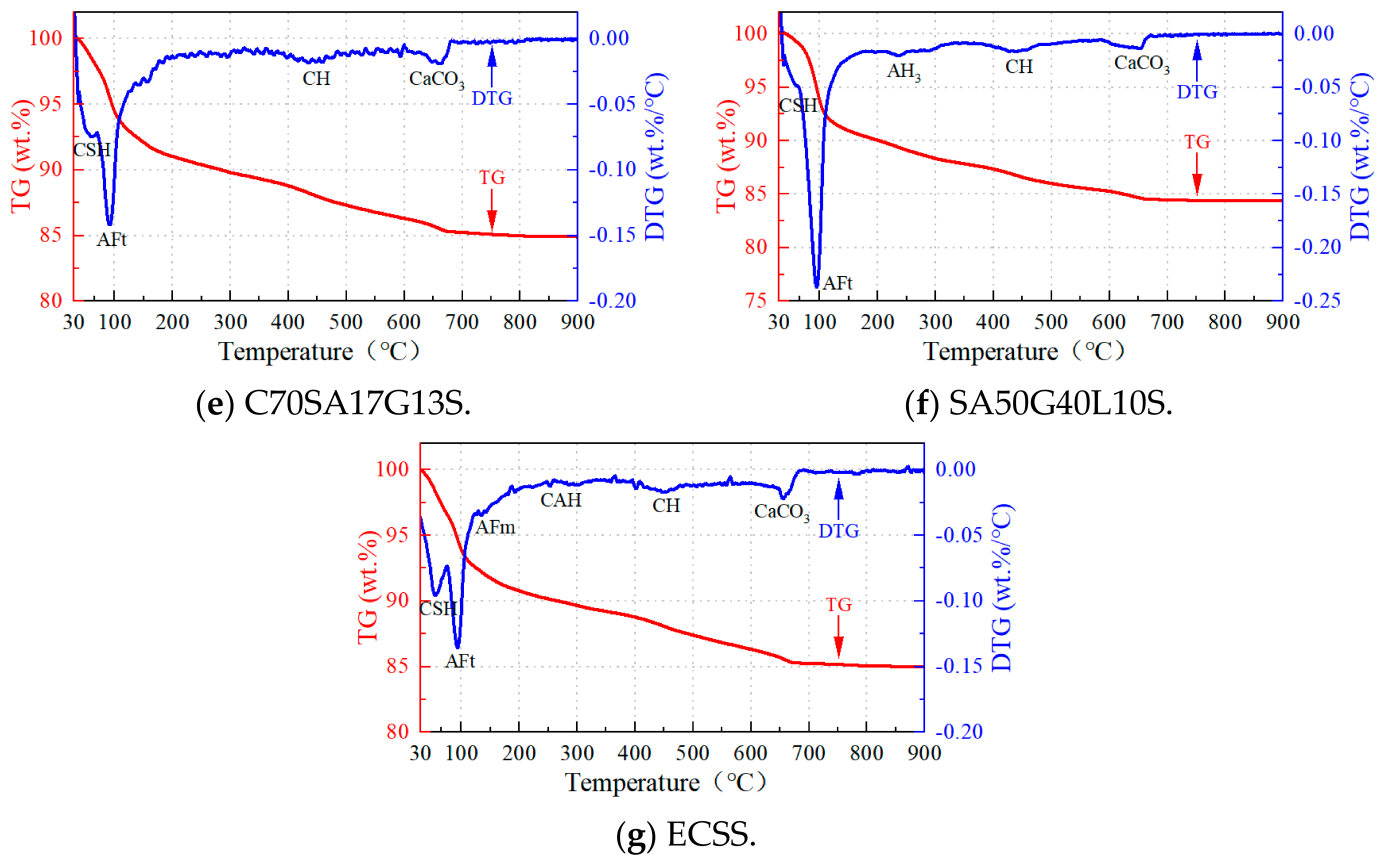
The microscopic test results of TG-DTG.

As can be seen from Figure 4a, C100S exhibits a strong peak of pore-adsorbed water release dominated by C–S–H gel at approximately 65 °C [12,34]. Faint peaks of crystal water release from AFt crystals and AFm crystals undergo changes at roughly 90 °C and 145 °C, respectively [35], and faint peaks corresponding to the decomposition and dehydration of CH crystals and the decomposition and decarbonation of CaCO_3_ at roughly 450 °C and 650 °C, respectively. An extremely faint peak corresponding to bound water release from CAH crystals may also be observed at approximately 260 °C [36].

Figure 4b shows that C70G30S exhibits a relatively strong peak of pore-adsorbed water release dominated by C–S–H gel at approximately 57 °C and an extremely strong peak of crystal water release from AFt crystals at around 98 °C, while no crystal water release peak associated with AFm crystals is observed.

In Figure 4d, C70SA25L5S exhibits a weak peak of pore-adsorbed water release dominated by C–S–H gel at approximately 77 °C, a minor thermal peak attributed to the crystal water dehydration of AFm phases occurs at 139 °C, and faint peaks associated with the decomposition and water loss of CH crystals and the decomposition and decarbonation of CaCO_3_ occur at roughly 450 °C and 650 °C, respectively. An extremely faint peak corresponding to bound water release from CAH crystals may also be present at approximately 260 °C. As can be seen from Figure 4e, C70SA17G13S exhibits a relatively strong peak of pore-adsorbed water release dominated by C–S–H gel at approximately 60 °C and a strong peak of crystal water release from AFt crystals at around 92 °C.

As shown in Figure 4f, SA50G40L10S exhibits a faint peak of pore-adsorbed water release dominated by C–S–H gel and a prominent crystal water loss peak release from AFt crystals at approximately 57 °C and 98 °C, respectively, while no AFm-related crystal water release peak is observed. In Figure 4g, ECSS exhibits a relatively strong peak of pore-adsorbed water release dominated by C–S–H gel at approximately 55 °C and a strong peak of crystal water release from AFt crystals and a weak crystal water loss peak release from AFm crystals at around 94 °C and 135 °C, respectively, as well as faint peaks associated with the decomposition and dehydration of CH crystals and the decomposition and decarbonation of CaCO_3_ at roughly 450 °C and 650 °C, respectively. An extremely faint peak corresponding to bound water release from CAH crystals may also be observed at approximately 260 °C.

#### 3.2.4. FTIR Results

The FTIR uses an effective microscopic technique to characterize the functional groups and chemical bond structures of materials. The absorption spectra collected at different wavenumbers can be adopted to identify organic and inorganic functional groups based on the position, intensity, and shift of characteristic absorption peaks. The changes in spectral peaks can reflect the formation of hydration products and the transformation of functional groups [37].

**Figure 5:**
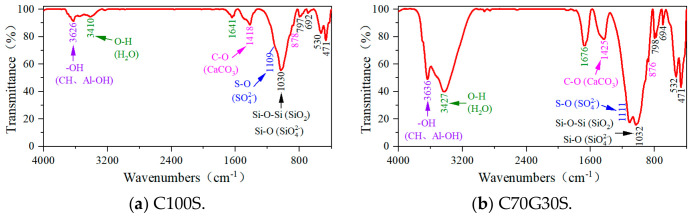

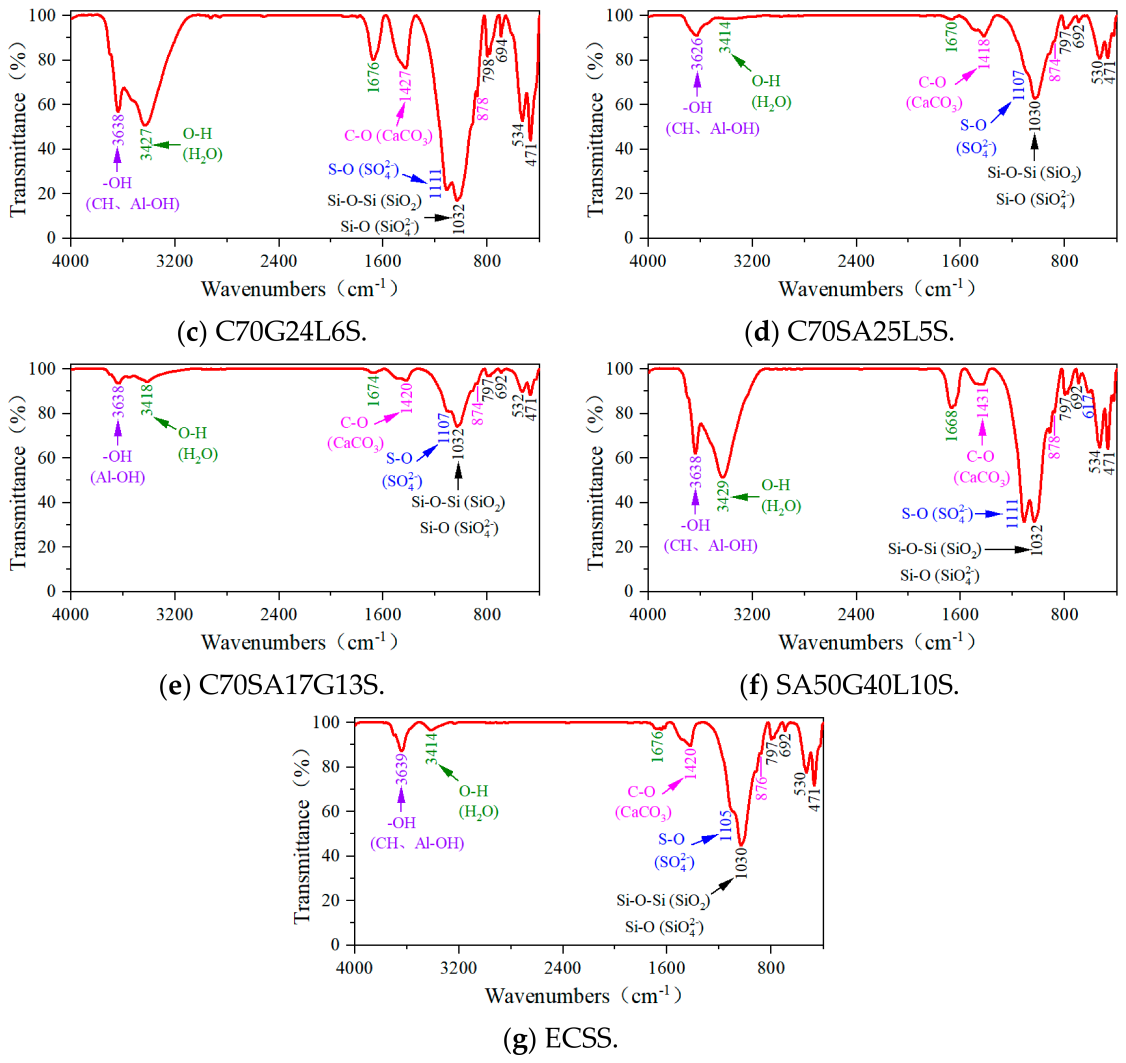
The microscopic test results of FTIR.

As can be seen from Figure 5a, the main component of the stabilized soil is still the soil itself. Therefore, the test results of all samples contain the absorption bands of Si-O-Si tetrahedrons in quartz, as well as the absorption bands of Si-O (from SiO_4_^2−^ tetrahedral anions) and Al-OH (from Al-OH octahedrons) in kaolin. These absorption bands are mainly reflected in the following aspects: a strong absorption band of asymmetric stretching vibration (resulting from the overlap of Si-O-Si bonds in quartz and Si-O bonds of SiO_4_^2−^ ions in kaolin) at a wavenumber of approximately 1030 cm^−1^; two split weak absorption bands of symmetric stretching vibration of Si-O-Si in quartz at around 797 cm^−1^ and 780 cm^−1^; a weak absorption band of asymmetric bending vibration of Si-O-Si in quartz at roughly 692 cm^−1^; a weak absorption band of symmetric bending vibration of Si-O-Si in quartz at about 471 cm^−1^; a weak absorption band of asymmetric bending vibration of Si-O (from SiO_4_^2−^ ions) in kaolin at approximately 530 cm^−1^; and an absorption band of stretching vibration of -OH (from Al-OH) in kaolin at around 3626 cm^−1^ [31,38,39]. Similar absorption features observed in the FTIR spectra of other stabilized soils are not repeated here.

Figure 5b shows that C70G30S exhibits an extremely strong absorption band of S-O (from SO_4_^2−^ ions) at a wavenumber of approximately 1111 cm^−1^, extremely strong absorption bands of O-H (resulting from the release of structural crystal water and interstitial adsorbed water in C–S–H gels, AFt crystals, and gypsum phases) at around 3427 cm^−1^ and 1676 cm^−1^, respectively, a relatively strong absorption band of -OH (from CH crystals) at roughly 3636 cm^−1^, and relatively strong absorption bands of CO_3_^2−^ ions (from CaCO_3_) at about 1425 cm^−1^ and 876 cm^−1^.

As depicted in Figure 5c, C70G24L6S soil exhibits an extremely strong absorption band of S-O (from SO_4_^2−^ ions) at a wavenumber of approximately 1111 cm^−1^, extremely strong absorption bands of O-H (from crystal water and pore-adsorbed water in C–S–H gels and AFt crystals) at around 3427 cm^−1^ and 1676 cm^−1^, respectively, a relatively strong absorption band of -OH (from CH crystals) at roughly 3638 cm^−1^, and relatively strong absorption bands of CO_3_^2−^ ions (from CaCO_3_) at about 1427 cm^−1^ and 878 cm^−1^. From the perspective of FTIR results, there is little difference between C70G24L6S, C70G30S, and G80L20S. A comprehensive analysis combined with SEM, XRD, and TG-DTG is still required.

In Figure 5d, C70SA25L5S exhibits a weak absorption band of S-O (from SO_4_^2−^ ions) at a wavenumber of approximately 1107 cm^−1^, weak absorption bands of O-H (resulting from the release of crystal structural water and interstitial adsorbed water in C–S–H gels and AFm crystal structures) at around 3414 cm^−1^ and 1670 cm^−1^, respectively, a relatively strong absorption band of -OH (from CH crystals) at roughly 3626 cm^−1^, and relatively strong absorption bands of CO_3_^2−^ ions (from CaCO_3_) at about 1418 cm^−1^ and 874 cm^−1^.

As can be seen from Figure 5e, C70SA17G13S exhibits a strong absorption band of S-O (from SO_4_^2−^ ions) at a wavenumber of approximately 1107 cm^−1^ and extremely strong absorption bands of O-H (resulting from the release of structural water and interstitial adsorbed water present in C–S–H gels and AFt crystal structures) at around 3418 cm^−1^ and 1674 cm^−1^, respectively.

The SA50G40L10S exhibits an extremely strong absorption band of S-O (from SO_4_^2−^ ions) at a wavenumber of approximately 1111 cm^−1^, extremely strong absorption bands of O-H (resulting from the release of crystal structural water and interstitial adsorbed water in C–S–H gels and AFm crystal structures) at around 3429 cm^−1^ and 1668 cm^−1^, respectively, a relatively strong absorption band of -OH (from CH crystals) at roughly 3638 cm^−1^, and relatively strong absorption bands of CO_3_^2−^ ions (from CaCO_3_) at about 1431 cm^−1^ and 878 cm^−1^ (Figure 5f).

In Figure 5g, the ECSS exhibits a relatively strong absorption band of S-O (from SO_4_^2−^ ions) at a wavenumber of approximately 1105 cm^−1^, weak absorption bands of O-H (resulting from the release of crystal structural water and interstitial adsorbed water in C–S–H gels and AFm crystal structures) at around 3414 cm^−1^ and 1676 cm^−1^, respectively, a weak absorption band of -OH (from CH crystals) at roughly 3639 cm^−1^, and weak absorption bands of CO_3_^2−^ ions (from CaCO_3_) at about 1420 cm^−1^ and 876 cm^−1^.

## 4. Discussion

The hydration products of C100S are mainly C–S–H gels (Figure 2a and Figure 5a). Its main reactive substances are C_3_S (accounting for approximately 50%) and C_2_S (accounting for approximately 25%) in cement, as shown in Equations (1) and (2):

2C_3_S + 6H → C_3_S_2_H_3_ + 3CH (1)

2C_2_S + 4H → C_3_S_2_H_3_ + CH (2)

**Figure 6:**
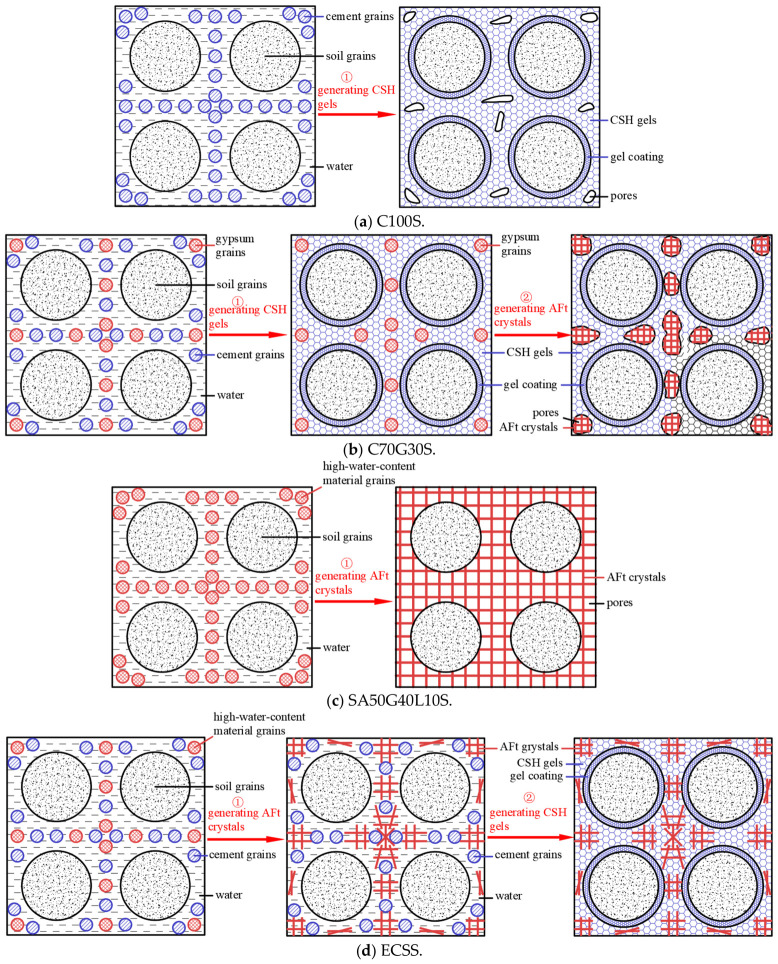
The structure formation models of the stabilized soils.

The SEM image (Figure 2a) shows that a large amount of C–S–H gel covers the outer surface of soil grains and binds the soil grains together. Some larger pores contain certain AFt crystals, AFm crystals, CH crystals, CAH crystals, and CaCO_3_ (Figure 2a, Figure 4a, Figure 5a and Figure 6a). C100S gains its major strength from C–S–H gels, AFt crystals, and calcium carbonate. Meanwhile, AFm, CH, and CAH phases barely contribute to the overall strength. Collectively, the composition of the hydration products and the microscopic structure of C100S are responsible for its excellent mechanical strength. The hydration products of C70G30S are still dominated by C–S–H gel (Figure 2b and Figure 5b), along with a large number of thick and elongated AFt crystals (Figure 2b, Figure 4b, Figure 5b and Figure 6b). Gypsum reacts with C_3_A and C_4_AF in cement to yield AFt crystals, as shown in Equations (3) and (4):

C_3_A + 6H → C_3_AH_6_ (3)

C_4_AF + 2CH + 10H → C_3_AH_6_ + C_3_FH_6_ (4)

Microscopic morphology observations indicate that excessive thick and long AFt crystals increase the size of pores among soil grains, while C–S–H gel fails to bind soil grains and AFt crystals together. As a result, the stabilized soil becomes loose (Figure 2b). Therefore, its strength is slightly lower than that of the C100S (Figure 1).

The hydration products of C70G24L6S and C70G30S are almost identical (Figure 2b,c, Figure 3(b,c), Figure 4b,c and Figure 5b,c). However, with the addition of lime, a pozzolanic reaction occurs [40], which promotes cement hydration and increases the generation rate of AFt crystals. The formation quantity of C–S–H gels is marginally elevated (Figure 4b,c), while the grain size of AFt crystals is effectively refined (Figure 2b,c). Microscopic morphology observations indicate that the refined AFt crystals infiltrate and fill the large intergranular pores of soil, forming a spatial network structure that differs greatly from the bulky AFt crystals in conventional cement–gypsum-stabilized soil. They do not cause an increase in the pores between soil grains; thus, the number of large pores decreases. In addition, a sufficient amount of C–S–H gel wraps around the outer surfaces of soil grains and the AFt crystal network spatial structure, and the cementation effect of C–S–H gel enables soil grains and the AFt crystal network spatial structure to form a relatively complete integrated mass (Figure 2c). Therefore, the strength of C70G24L6 is significantly greater than that of C70G30S (Figure 1) [17].

In addition, when comparing C70G24L6S with C100S, although the amount of C–S–H gel in the former is slightly reduced (Figure 4a,c), the number of large pores is substantially reduced due to pore filling by smaller AFt crystals (Figure 2a,c). Ultimately, the strength of C70G24L6S is slightly greater than that of C100S (Figure 1). Therefore, variations in the microstructure of AFt crystals constitute a major determinant of strength development in calcium-reinforced soil systems [17].

The hydration products of C70SA25L5S are dominated by C–S–H gel (Figure 2d and Figure 4d; Equations (1) and (2)) and contain small amounts of AFm crystals, CH crystals, CAH crystals, and CASH gel.

In C70SA17G13S, a larger number of AFt crystals are formed, while no CAH or AFm crystals are observed (Figure 2e, Figure 3(e), Figure 4e and Figure 5e). All CAH undergoes a secondary reaction with gypsum to form AFt (Equation (5)). The hydration products of C70SA17G13S are therefore almost identical to those of C70G24L6S, being dominated by C–S–H gel and AFt crystals. The smaller AFt crystals fill the large pores between soil grains to form a network structure, while C–S–H gel wraps around the outer surfaces of the soil grains and the AFt network structure, forming a relatively dense and coherent integrated mass (Figure 2c,e, Figure 3(c,e), Figure 4c,e and Figure 5c,e). Consequently, the strength of C70SA17G13S is comparable to that of C70G24L6S (Figure 1).

(5) $$3C\bar{S}+C_{3}{\mathrm{AH}}_{6}+26H\to C_{6}A{\bar{S}}_{3}H_{32}$$

The hydration products of SA50G40L10S are dominated by an extremely large number of small AFt crystals (Figure 2f, Figure 3(f), Figure 4f and Figure 5f; Equations (5) and (6)) and contain trace amounts of C–S–H gel (Figure 4f; Equation (2)). Microscopic morphology observations indicate that these small AFt crystals can fill the pores between soil grains; however, the cementation capacity of AFt crystals is relatively poor, causing the entire sample to become relatively loose (Figure 2f). AFt crystals are therefore the primary contributors to the strength of SA50G40L10S. Although the intrinsic strength of AFt crystals is moderate, the expansive curing agent exhibits an extremely strong water-fixing capacity (Equations (5) and (6)). Consequently, the strength of SA50G40L10S is lower than that of cement-stabilized soil (Figure 1).

The hydration products of ECSS are dominated by C–S–H gel (Equations (1) and (2)) and AFt crystals (Equations (5) and (6)) and contain trace amounts of AFm crystals, CASH gel, CH crystals, and CaCO_3_ (Figure 2e, Figure 3(e), Figure 4e and Figure 5e). The formation of AFt exhibits an extremely strong water-fixing capacity (Equations (5) and (6)). C–S–H gel phases and AFt crystal networks are the major mechanical contributors responsible for the strength performance of ECSS. AFt crystals infiltrate the coarse pores of soil particles and develop an interconnected three-dimensional network, while the cementation effect of C–S–H gel enables soil grains and the AFt network structure to form a highly dense and coherent integrated mass. The functional roles of both C–S–H gel and AFt crystals are therefore fully mobilized, achieving an optimal synergistic combination. As a result, the strength of ECSS is the highest among all stabilized soils investigated in this study (Figure 1).

(6) $$C_{4}A_{3}\bar{S}+2C\bar{S}+38H\to C_{6}A{\bar{S}}_{3}H_{32}+2{\mathrm{AH}}_{3}$$

## 5. Structure Formation Models of the Stabilized Soils

Based on the microstructural morphology, main hydration products, hydration reaction processes, and reaction rates of the stabilized soils analyzed in the micro-performance study presented in Section 3, the structure formation models for C100S, C70G30S, SA50G40L10S, and ECSS can be proposed, as shown in Figure 6.

After the completion of the hydration reaction of C100S, its main hydration product is C–S–H gel. The C–S–H gel coats the outer surfaces of soil grains and fills the pores between the grains. However, some relatively large pores still remain, and the stabilized soil exhibits relatively high strength.

The hydration reaction of C70G30S occurs in two stages. The first stage is the hydration reaction of cement, which produces C–S–H gel and CAH. The second stage involves the reaction between gypsum and CAH to form AFt crystals. Due to the expansive nature of AFt crystals, the thick and elongated AFt crystals formed in the later stage expand the small pores between soil grains to larger sizes and partially damage the hardened structure of the original C–S–H gel. Moreover, there is no subsequent formation of C–S–H gel to coat the AFt crystals and fill these newly formed pores. Therefore, the strength of C70G30S is slightly lower than that of C100S.

After the completion of the hydration reaction of SA50G40L10S, its main hydration product is AFt crystals. Although AFt crystals can fill the pores between soil grains, their cementation capacity is relatively poor. Consequently, the strength of SA50G40L10S is lower than that of C100S.

The hydration reaction of ECSS takes place in two steps: The first step involves the rapid hydration reaction of materials such as sulfur aluminate cement, gypsum, and lime, which generates AFt crystals and fixes a large amount of free water. This process fills the large pores between soil grains, forming a three-dimensional network structure. The second stage is the formation of C–S–H gel from cement, which proceeds through a relatively slow hydration reaction. The cementation effect of the C–S–H gel enables the soil grains and the AFt crystal network structure to form a highly integrated and coherent whole. As a result, the strength of ECSS is significantly higher than that of C100S.

In the C70G30S, the prerequisite for AFt crystal formation is the presence of CAH, which originates from cement hydration reactions; therefore, the formation time of AFt crystals must necessarily be later than that of C–S–H gel. In the ECSS, the reaction of sulfur aluminate cement is much faster than that of Portland cement, resulting in the formation of AFt crystals occurring earlier than C–S–H gel. The sequence of formation between C–S–H gel and AFt crystals in the two formulations affects the microstructure of high-water-content stabilized soils, thereby influencing their strength.

**Figure 7:**
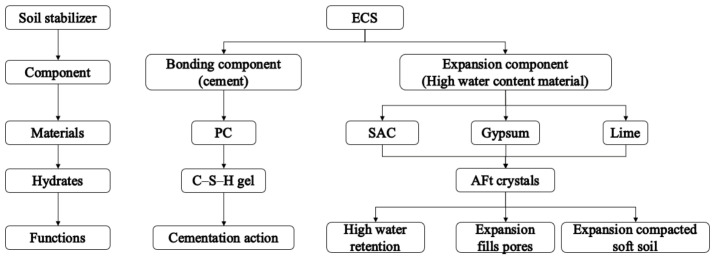
The compositions of the expansible–cementitious stabilizer (ECS).

According to the representative tests of stabilizers for soft soil with high water content, ECSS exhibits the highest strength. In addition, the stabilization mechanism of ECSS is revealed by combining multiple microstructural testing methods. Its hydration products are mainly composed of C–S–H gel (with strong cementation capability) and AFt crystals (providing high water stabilization, expansive pore filling, and soft soil compaction). The components of ECS consist of two parts: a cementitious component and an expansive component, as shown in Figure 7.

(1) Cementitious component

The raw material in this component is ordinary Portland cement, whose function is to generate C–S–H gel with cementitious properties through hydration reactions. The C–S–H gel bonds soil grains and other hydration products together to form a dense and coherent integrated structure. Owing to its high intrinsic strength, C–S–H gel is the primary source of strength in ECSS. This conclusion can be extended to all cementitious materials capable of generating C–S–H gel.

(2) Expansive component

The raw materials in this component include sulfur aluminate cement clinker, gypsum, and lime. The optimal mass ratio of these three materials is 5:4:1 [19,41], and this ratio is adopted throughout this study for the expansive component of ECS. The primary function of this component is to generate AFt crystals through hydration reactions, with a formation rate significantly higher than that of C–S–H gel [19]. AFt crystals contain 32 molecules of crystallization water; during hydration, they can bind a large amount of pore water in soft soil. Their solid-phase volume can increase by approximately 125%, and their formation rate is much faster than that of C–S–H gel. AFt crystals are predominantly acicular and columnar in morphology. Consequently, the expansive component provides high water stabilization, expansive pore filling, and soft soil compaction, making it highly suitable for stabilizing soft soils with high water content [18].

In summary, the rapid formation of AFt crystals absorbs a large amount of water in high-water-content soft soil at an early stage and fills large intergranular pores to form a three-dimensional network structure, thereby establishing the basis for early strength development. At later stages, the cementation effect of C–S–H gel integrates soil grains with the AFt network structure into a highly coherent composite system. The synergistic functions of C–S–H gel and AFt crystals are thus fully mobilized, achieving an optimal stabilization mechanism [17].

## 6. Conclusions

This study systematically investigated the stabilization behavior of high-water-content soft soil using ordinary Portland cement, sulfur aluminate cement, gypsum, and lime through a series of controlled laboratory experiments. The following are some major conclusions:

- Stabilized soils containing cementitious components exhibit significantly higher compressive strength than those without cementitious component, demonstrating that the cementation effect of C–S–H gel is the primary mechanism governing strength enhancement in high-water-content soft soils.
- By incorporating expansive components (i.e., sulfur aluminate cement, gypsum, and lime) with cement in appropriate proportions, a new composite stabilizer (ECS) suitable for high-water-content soft soils was proposed in this study. The compressive strength of ECS-stabilized soil is 162.18% of that of cement-stabilized soil. The expansive components predominantly generate ettringite (AFt) crystals during hydration, whereas the cementitious component mainly produces calcium silicate hydrate (C–S–H) gel.
- The structure formation model of ECS-stabilized soil is as follows: The rapid formation of AFt crystals in the early stage effectively absorbs excess pore water and fills large intergranular pores, creating a three-dimensional network structure that provides the basis for early strength development. At later stages, the cementation effect of C–S–H gel integrates soil particles with the AFt crystal network, resulting in a dense and coherent composite structure and thereby ensuring sustained strength gain.
- The effects of curing age, additive dosage, cement replacement rate, soil type, and construction factors on the mechanical properties, workability, and durability of ECS-stabilized soil still require further investigation.

## Figures and Tables

**Table 1:** Oxide compositions of the tested soil and stabilizer components.

The Oxide Compositions ^①^	Mass Percentage (%)				
	Soil	C	L	G	SA
SiO_2_	64.47	19.83	0.28	1.38	11.29
Al_2_O_3_	15.86	7.75	0.13	0.08	27.84
CaO	1.22	52.42	73.83	35.85	42.63
SO_3_	0.02	3.70	0.24	45.59	8.52
Fe_2_O_3_	5.72	3.97	0.28	0.03	2.06
MgO	1.32	2.25	0.50	3.38	1.32
K_2_O	2.14	0.77	0.01	—	0.27
TiO_2_	0.80	0.34	—	—	1.00
Na_2_O	0.55	0.18	—	—	0.21
Others	0.03	0.31	0.01	0.39	0.22
LOI ^②^	7.87	8.48	24.72	13.30	4.64

Note: ① The oxide composition was determined using an XRF spectrometer (Bruker S8 TIGER, Ettlingen, Germany). ② The LOI was determined at 950 °C.

**Table 2:** Grouping details of the stabilizer representative tests.

Stabilizer Type	Number	Mass Percentage of Each Component in the Stabilizer (%)			
		C	L	G	SA
Single mixed	C100S	100	—	—	—
	L100S	—	100	—	—
	G100S	—	—	100	—
	SA100S	—	—	—	100
Binary mixed	C70L30S	70	30	—	—
	C70G30S	70	—	30	—
	C70SA30S	70	—	—	30
	G80L20S	—	20	80	—
	SA83L17S	—	16.7	—	83.3
	SA56G44S	—	—	44.4	55.6
Ternary mixed	C70G24L6S	70	6	24	—
	C70SA25L5S	70	5	—	25
	C70SA17G13S	70	—	13.3	16.7
	SA50G40L10S	—	10	40	50
Quaternary mixed	C70SA15G12L3S (ECSS)	70	3	12	15

**Table 3:** Test and measurement methods of the stabilized soils.

Stabilizer Type	Number	Methods				
		UCS	SEM	XRD	TG-DTG	FTIR
Single mixed	C100S	√	√	√	√	√
	L100S	√	×	×	×	×
	G100S	√	×	×	×	×
	SA100S	√	×	×	×	×
Binary mixed	C70L30S	√	×	×	×	×
	C70G30S	√	√	√	√	√
	C70SA30S	√	×	×	×	×
	G80L20S	√	×	×	×	×
	SA83L17S	√	×	×	×	×
	SA56G44S	√	×	×	×	×
Ternary mixed	C70G24L6S	√	√	√	√	√
	C70SA25L5S	√	√	√	√	√
	C70SA17G13S	√	√	√	√	√
	SA50G40L10S	√	√	√	√	√
Quaternary mixed	C70SA15G12L3S (ECSS)	√	√	√	√	√

Note: “√” indicates the tests that have been conducted; “×” indicates the tests that have not been conducted.

## Data Availability

The original contributions presented in this study are included in the article. Further inquiries can be directed to the corresponding author.

## References

[1] Xu R.Q., Zhang B.L., Zhang G.P. (2024). Research progress of soft soil stabilizer. J. Ground Improv..

[2] Dai F.Z. (2024). Research progress on dynamic characteristics of solidified soil. China Water Transp..

[3] Ting W.X., Yi Q., Bin C. (2021). Solidification effect and mechanism of ionic soil stabilizer applied on high-water-content clay. Bull. Eng. Geol. Environ..

[4] Li W.T., Yi Y.L., Puppala A.J. (2022). Effects of curing environment and period on performance of lime-GGBS-treated gypseous soil. Transp. Geotech..

[5] Banfill P.F.G. (2006). Alkali-activated cements and concretes. Adv. Cem. Res..

[6] Jia Z., Yang Y., Yang L., Zhang Y., Sun Z. (2018). Hydration products, internal relative humidity and drying shrinkage of alkali activated slag mortar with expansion agents. Constr. Build. Mater..

[7] Kim M.S., Jun Y., Lee C., Oh J.E. (2013). Use of CaO as an activator for producing a price-competitive non-cement structural binder using ground granulated blast furnace slag. Cem. Concr. Res..

[8] Sharma A.K., Sivapullaiah P.V. (2016). Swelling behaviour of expansive soil treated with fly ash–GGBS based binder. Geome-Chanics Geoengin..

[9] Yu W., Zhu Z.D., Zhang D.W., Sun H., Li Y. (2024). Research on strength and deformation properties of high moisture soft soils stabilized by GGBS, cement, fly ash, and sodium silicate. Constr. Build. Mater..

[10] Li X., Wang Y., Zhang H. (2024). Synergistic effects of recycled demolition waste and GGBS-FA based geopolymers on the mechani-cal properties and stabilization mechanism of high plasticity clay. Case Stud. Constr. Mater..

[11] Samaptika M., Nagendra R. (2021). Strength and durability of flyash, GGBS and cement clinker stabilized dispersive soil. Cold Reg. Sci. Technol..

[12] Jiang N.J., Du Y.J., Cai K. (2018). Durability of lightweight alkali-activated ground granulated blast furnace slag (GGBS) stabilized clayey soils subjected to sulfate attack. Appl. Clay Sci..

[13] Hou W.J., Ye F., Yu Y., Chen D., Wang J., Liu F., Feng D., Liang S. (2025). Experimental study on the strength characteristics and seawater degradation resistance of sandy silt solidified with alkali-activated slag. Constr. Build. Mater..

[14] Zhou Y., Huo M., Zhang L. (2024). Strength development and solidification mechanism of soils with different properties stabilized by cement-slag-based materials. Case Stud. Constr. Mater..

[15] Liu J., Zhao B., Zhang S. (2024). Experimental study on preparation of solidified soil from residue of construction waste. Constr. Build. Mater..

[16] Huang X., Hu T.A. (1998). On stabilization of soft soil with waste gypsum and cement. Chin. J. Geotech. Eng..

[17] Han Y.M., Xia J.W. (2021). The Influence Mechanism of Ettringite Crystals and Microstructure Characteristics on the Strength of Calcium-Based Stabilized Soil. Materials.

[18] Yan Z.P., Yang H.Y., Zhu Z.L. (1999). Feasibility study on the application of high-water quick-setting material in soft soil foundation treatment. Guang Dong Gong Lu JiaoTong.

[19] Sun H.H., Yan F.X. (1992). The performance and application technology of high-water quick-setting filling material. China Energy Environ. Prot..

[20] (2019). Standard for Geotechnical Testing Method.

[21] MolaAbasi H., Semsani S.N., Saberian M., Khajeh A., Li J., Harandi M. (2020). Evaluation of the long-term performance of stabilized sandy soil using binary mixtures: A micro- and macro-level approach. J. Clean. Prod..

[22] Liang S., Chen J., Guo M. (2020). Utilization of pretreated municipal solid waste incineration fly ash for cement-stabilized soil. Waste Manag..

[23] Choobbasti A.J., Kutanaei S.S. (2017). Microstructure characteristics of cement-stabilized sandy soil using nanosilica. J. Rock Mech. Geotech. Eng..

[24] Yang S., Liu W. (2019). The Effect of Changing Fly Ash Content on the Modulus of Compression of Stabilized Soil. Materials.

[25] Ahmed A. (2015). Compressive strength and microstructure of soft clay soil stabilized with recycled bassanite. Appl. Clay Sci..

[26] Li J.P., Li T. (2020). Stability Mechanism and Research Progress of Soil Stabilizer. Mater. Rep..

[27] Ding J.W., Zhang S., Hong Z.S. (2010). Experimental study of stabilization of dredged clays with high water content by adding cement and phosphogypsum synchronously. Rock Soil Mech..

[28] Chen Y. (2017). A Study of Solidified Mechanism and Effects of Solidified Agent of Reinforcing Soft Soil. J. Anhui Univ. Sci. Technol. (Nat. Sci.).

[29] Maichin P., Jitsangiam P., Nongnuang T., Boonserm K., Nusit K., Pra-Ai S., Binaree T., Aryupong C. (2021). Stabilized High Clay Content Lateritic Soil Using Cement-FGD Gypsum Mixtures for Road Subbase Applications. Materials.

[30] (2021). X-Ray Powder Diffraction Analysis for Phase Determination of Portland Cement Clinker.

[31] Sharma L.K., Sirdesai N.N., Sharma K.M., Singh T.N. (2018). Experimental study to examine the independent roles of lime and cement on the stabilization of a mountain soil: A comparative study. Appl. Clay Sci..

[32] Zhang H., Wang L., Long H.M. (2018). Study on Composite Activating Mechanism of Alkali Steel Slag Cementations Materials by XRD and FTIR. Spectrosc. Spectr. Anal..

[33] (2017). Methods for Chemical Analysis of Cement.

[34] Du Y., Yu B., Liu K. (2016). Physical, Hydraulic, and Mechanical Properties of Clayey Soil Stabilized by Lightweight Alka-li-Activated Slag Geopolymer. J. Mater. Civ. Eng..

[35] Jha A.K., Sivapullaiah P.V. (2016). Gypsum-Induced Volume Change Behavior of Stabilized Expansive Soil with Fly Ash-Lime. Geotech. Test. J..

[36] Jiang N., Du Y., Liu S. (2016). Multi-scale laboratory evaluation of the physical, mechanical, and microstructural properties of soft highway subgrade soil stabilized with calcium carbide residue. Can. Geotech. J..

[37] (2019). General Rules for Infrared Spectrometric Analysis.

[38] Zhang T., Cai G., Liu S. (2018). Application of Lignin-Stabilized Silty Soil in Highway Subgrade: A Macroscale Laboratory Study. J. Mater. Civ. Eng..

[39] Wei L., Chai S., Guo Q. (2020). Mechanical properties and stabilizing mechanism of stabilized saline soils with four stabilizers. Bull. Eng. Geol. Environ..

[40] Guo Y. (2007). Study on Stabilization of Muddy Soil Mechanical Properties of Stabilized Soil. Ph.D. Thesis.

[41] Xia J.W., Su Q., Liu D.D. (2018). Optimal gypsum-lime content of high water material. Mater. Lett..

